# General work stress and suicide cognitions in health-care workers: mediating effect of hopelessness and job satisfaction

**DOI:** 10.3389/fpubh.2023.1254331

**Published:** 2023-10-17

**Authors:** Abdulselami Sarigül, Alican Kaya, Izaddin Ahmad Aziz, Murat Yıldırım, Halil Ibrahim Özok, Francesco Chirico, Salvatore Zaffina

**Affiliations:** ^1^Department of Therapy and Rehabilitation, Ağrı İbrahim Çeçen University, Ağrı, Türkiye; ^2^Department of Guidance and Psychological Counselling, Ağrı İbrahim Çeçen University, Ağrı, Türkiye; ^3^Special Education Department, College of Education, Salahaddin University, Erbil, Iraq; ^4^Department of English, College of Education, Bayan University, Erbil, Iraq; ^5^Faculty of Science and Letters, Department of Psychology, Ağrı İbrahim Çeçen University, Ağrı, Türkiye; ^6^Graduate Studies and Research, Lebanese American University, Beyrut, Lebanon; ^7^Department of Measurement and Assessment, Van Yüzüncü Yıl University, Van, Türkiye; ^8^Post-Graduate School of Occupational Health, Università Cattolica del Sacro Cuore, Rome, Italy; ^9^Health Service Department, Italian State Police, Ministry of the Interior, Milan, Italy; ^10^Occupational Medicine/Health Technology Assessment and Safety Research Unit, Clinical-Technological Innovations Research Area, Bambino Gesù Children’s Hospital, IRCCS, Rome, Italy

**Keywords:** general work stress, suicide cognitions, hopelessness, job satisfaction, health-care workers

## Abstract

Individuals with a satisfactory level of job satisfaction are much less likely to feel hopeless about their future and are more likely to perform efficiently in the workplace. General work stress (i.e., the work-related stress subjectively experienced) is a significant predictor of suicide cognitions. Furthermore, it has been posited that satisfaction and hope are fundamental to life from an existential perspective. We, therefore, tested a hypothetical model of general work stress, suicide cognitions, hopelessness, and job satisfaction. The data were collected from 416 health-care workers through a convenience sampling method. The mediation analysis results revealed significant negative and positive relationships among general work stress, suicide conceptions, hopelessness, and job satisfaction. The findings indicate that hopelessness and job satisfaction have a parallel mediating effect in the relationship between general work stress and suicide cognitions. The result of the study is of great importance, which suggests that interventions to alleviate hopelessness and work stress and to boost the job satisfaction of medical staff may help prevent suicide cognitions.

## Introduction

Work stress refers to the mental and physical discomfort health-care staff in health-care workplaces experience because of their duties (1, 2). General work stress arises due to the interaction between employees and their work, negatively affecting mental and physical health, reducing the employee’s standard of living, and causing various work-related negativities (3). As well as having physical consequences such as behavioral disorders, there may also be mental consequences such as depression, burnout, anxiety, and suicidal thoughts (4, 5). A variety of types of stress may affect an employee’s performance at work, such as job stress, academic stress, environmental stress, health stress, relationship stress, and especially family stress (6–8).

Work stress can create intense pressure on health-care workers (9, 10). Several epidemiological studies have indicated that employees exposed to work stress may experience intense suicidal thoughts (11–14). An effort-reward imbalance (ERI) model describes the disparity between employee job pressure, the amount of effort they put into their jobs, and the low reward they get (15). Those who put forth great effort at work and perform tasks that risk their health will likely experience chronic work-related stress in the long run if the reward they receive is not commensurate with their effort (16). Employees may ultimately realize their thoughts are hurting them, resulting in suicidal thoughts (17, 18). It is essential to disclose other risk factors that may lead to suicide cognitions in health-care workers to prevent suicide (19). Health-care workers have been found to have higher suicide cognitions than the general population due to work stress (20–22).

The work stress in health-care staff can be associated with various mental health problems (e.g., depression, anxiety, and stress) (23). According to Godifay et al. (24), health-care workers may be at greater risk for work stress than others, and it is closely linked to job satisfaction. In general, job satisfaction refers to a sense of well-being based on the profession’s role in society, the experience of the employee, and the ability to evaluate them as a professional (25). The level of job satisfaction has been reported to affect the quality and delivery of health-care services and mediate the relationship between patient health outcomes (26).

Job satisfaction is closely related to individuals’ emotional relationship with their work and the pleasure and dissatisfaction they feel while doing their work (27). The high job satisfaction of health-care workers reduces their work stress while also helping them to perform more effectively. (28, 29). A lack of job satisfaction results in reduced ability to be productive at work and problems with attendance and negativity, which may result in termination from the position (30). Health workers are more likely to increase their work productivity if they are satisfied with their jobs, and those with increased work stress are less likely to be satisfied with their jobs (31–33). Stress may result in a decrease in job satisfaction and an increase in mental health symptoms such as anxiety, depression, and thoughts of suicide (34–39). Among health-care workers, stress at work may have led to problems in personal relationships, concentration problems, and physical problems, leading to hopelessness (40, 41). In addition to work-related stress, personal isolation, the possibility of death, and fatigue caused by wearing protective clothing for an extended period of time may have contributed to hopelessness among health-care personnel (41–45).

Hopelessness has been associated with psychological concepts such as work-related stress, suicide, anxiety, conflict, and burnout (43, 46–48). This refers to emotional states in which the individual believes any attempt to affect change will be futile (49). The studies suggest that self-harm, anxiety, fear, anxiety, depression, and suicidal ideation are some negative outcomes that hopelessness might be related (50, 51). Suicide can result from an individual’s belief that they are unable to do something due to hopelessness (52, 53). Hopelessness theory suggests that individuals may develop a greater risk of suicidal ideation when they perceive themselves trapped in an impossible situation without a sense of escape or improvement (52). This may lead individuals to consider suicide a viable option to escape their pain if they do not have hope for the future.

## Aims and objectives

**Figure 1:**
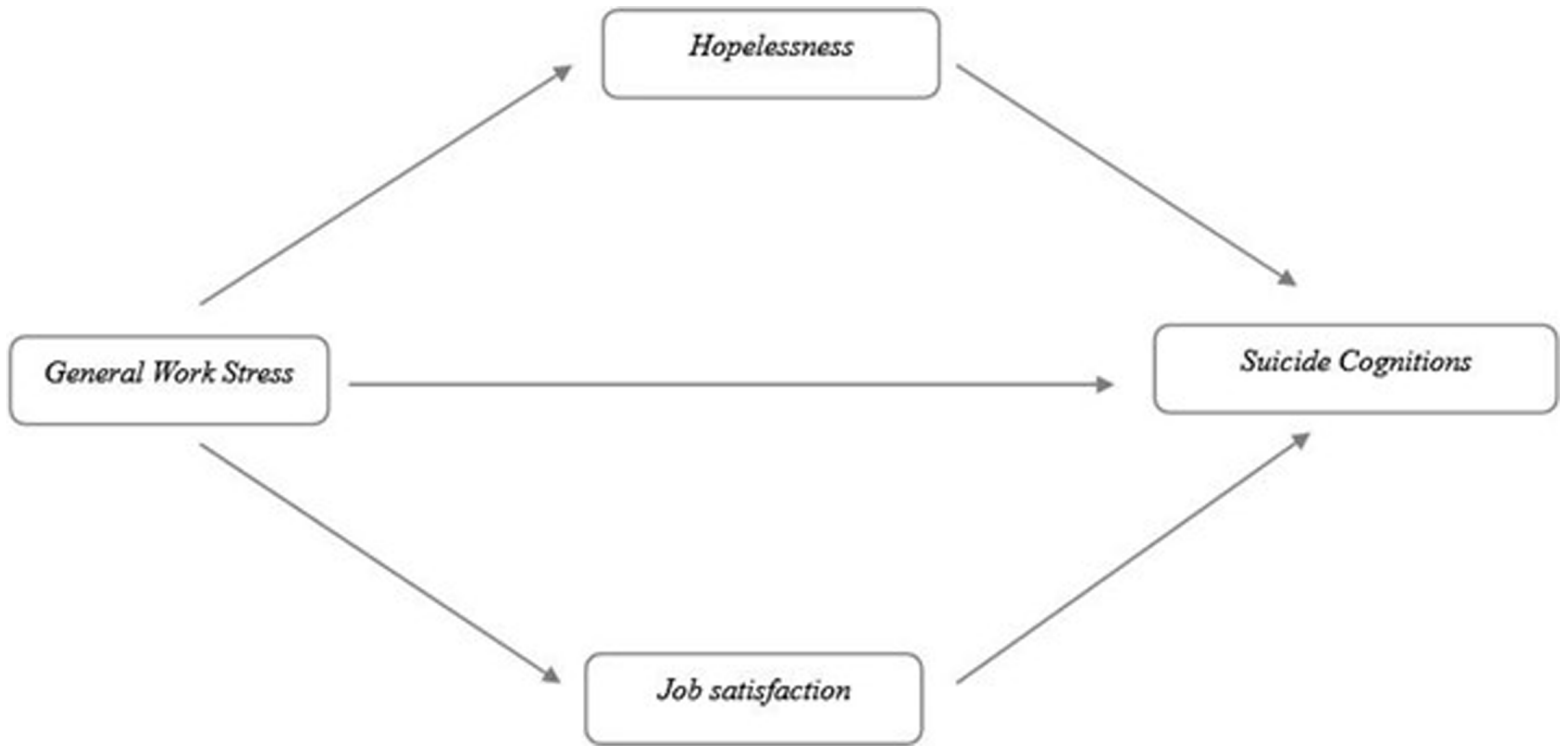
The proposed structural model.

This cross-sectional study is grounded in the framework of the Hopelessness Theory and Effort–Reward Imbalance. Previous empirical research has demonstrated that general work-related stress is a significant predictor of suicide cognitions (54, 55). Additionally, various studies have identified the relationships between general work stress, suicide cognitions, hopelessness, and job satisfaction (22, 32, 33, 47, 54, 56–58). In light of both theoretical foundations and empirical evidence, we propose a new model to investigate the relationships between the above-mentioned variables. This model examines the mediating effect of hopelessness and job satisfaction in the relationship between general work stress and suicide cognitions (see Figure 1). The following hypotheses were addressed in our study: (i) general work stress would have a significant negative impact on job satisfaction and a significant positive impact on hopelessness and suicide cognitions, (ii) hopelessness would serve as a mediating factor in the relationship between general work stress and suicide cognitions, and (iii) job satisfaction would serve as a mediator in the relationship between general work stress and suicide cognitions.

## Method

### Participants and ethics

Participants included 416 medical staff (70.9% female and 121 male) working in Türkiye. The age range of the participants was 21–60, with a mean age of 26.96 (SD = 7.16). They self-expressed themselves regarding their socioeconomic status (Low SES = 28.8%, Moderate SES = 64.9%, and High SES = 6.3%). Eligibility criteria included (i) being a health worker, (ii) working in any public or private health institution, (iii) participating voluntarily. Incentives were not provided to participants. The ethics committee at the university of the first author approved the study (reference number: E.73559). The study was conducted from October 2022 to May 2023.

### Power analysis

The power analysis was performed in order to reveal accurately and strongly the relationships between the predictor and predicted variable determined within the scope of the study. The analysis was conducted using the G* Power 3.1.9.7 program to determine the sample size required. Accordingly, with conventional significance levels of 0.05 and power of 0.80, a small effect size is defined as *r* = 0.20 (59). A total of 395 samples were required based on the results of the analysis. Upon reaching a sufficient sample size, the power analysis was repeated as a *post hoc* procedure under the same conditions. The power of the sample size of the study was calculated as 0.82 (1-*β* err probe). The results of this analysis indicate that the sample had a sufficient level of power for the analyses.

### Measures

*General Work Stress Scale* [GWSS: (60); Turkish version: (61)]. The GWSS was developed to measure one’s general work stress level. The scale includes 9 items (e.g.*, Have you ever lost your temper due to stress at work? or When you are stressed at work, do you forget to complete important tasks?*), and all items are rated on a 5-point Likert scale type ranging from 1 (*Never*) to 5 (*Every time*). The higher the score, the greater the level of general work stress. Cronbach’s α was 0.91, and McDonald’s ω was 0.91, in this study.

*Suicide Cognitions Scale* [SCS: (Rudd et al., unpublished)^1^; Turkish version: (62)]. The BRS was developed to measure one’s suicide cognitions. The scale includes 18 items (e.g.*, My only solution to my problems is to end my life. Or I would rather die right now than endure this unbearable pain*). The higher the score on the scale, the greater the level of suicide cognition. Cronbach’s α was 0.95, and McDonald’s ω was 0.95, in this study.

*Beck Hopelessness Scale* [BHS: (63, 64)]. The BHS was developed to measure one’s hopelessness level by using 20 items including true and false propositions (e.g.*, As I cannot change myself, it is best to stop trying. Or Even when something goes wrong, it is comforting to know that things will not always remain the same*). Higher scores on the scale indicate greater hopelessness. Cronbach’s *α* was 0.75, and McDonald’s *ω* was 0.75, in this study.

*Job Satisfaction Scale* [JSS: (65); Turkish version: (66)]. The BRS was developed to measure positive emotional state resulting from the subjective perception of the person’s work experiences. The scale includes 5 items ranging from 1 (*Strongly disagree*) to 5 (*Strongly agree*) (e.g.*, My job is enjoyable to me. Or My current job is very satisfying to me*). A higher score on the scale indicates, a higher level of suicide cognition. In this study, Cronbach’s *α* was 0.81, and McDonald’s *ω* was 0.81.

### Procedures

We followed the Declaration of Helsinki at all stages of the study. We used an online survey to collect data. The online survey provided participants with a brief explanation of the study’s purpose. Health-care professionals working at different hospitals in Turkey received an invitation text/email containing study information and an informed consent form. It provided information about the study, including its objectives and duration, assurances of anonymity and confidentiality, and voluntary participation in the study. Additionally, the survey is stated to be limited to one completion per participant. The questionnaires were administered only after informed consent had been obtained from the participants. Participants in the study were asked whether they would be willing to participate voluntarily. Those who indicated that they had not participated in the study voluntarily were not permitted to continue. There was a warning to participants that if they did not wish to fill out the questionnaires or if they did not feel comfortable, they could leave at any time during the research. Participants were eligible for inclusion if they were over 18 years of age, participating voluntarily, and were health workers. To avoid trust problems that may arise during the answering process on the scales, they have been asked not to enter their personal information into the online form. The confidentiality and anonymity of the responses were assured. Since the research subject was suicide cognitions, some participants did not want to complete questionnaires. We did not include in the study those participants who refused to participate.

### Data analysis

A number of assumptions, including multicollinearity and normality, were tested before the primary analysis was conducted. The skewness and kurtosis statistics were calculated in order to test the assumption of normality. The Variance inflation factor (VIF), Tolerance statistics, and Condition index have been computed to test the multicollinearity assumption. There should be a tolerance value of less than 0.2, a VIF value of less than 10, and a condition index of less than 15 (67, 68). A Mahalanobis distance was calculated to remove outliers, and it was determined that 23 participants should be removed from the data set as a result of the analysis. Outliers are often detected by using a technique called Mahalanobis distance (69, 70). After examining the preliminary analysis, we tested a parallel mediation model to determine whether hopelessness and job satisfaction mediated the relationship between general work stress and suicide cognitions. A confidence interval of 95% was used to explain the indirect effects of the proposed model (71). In order to test whether indirect effects were significant, bias-corrected bootstrapping procedures were applied. The bootstrap value was set to 10,000. We analyzed all of the data using SPSS 26.0 and used the R-based Jamovi 1.6.23 (The Jamovi Project, 2022) in conjunction with the jAMM module for the mediation analysis (72).

## Results

Table 1 presents participants’ demographic details, including means (M) and standard deviations (SD) for the variables. An independent sample *t*-test was performed to compare the general work stress, suicidal cognitions, hopelessness, and job satisfaction by gender, marital status, and Covid-19 experience. General work stress, suicidal cognitions, hopelessness, and job satisfaction did not differ significantly based on gender and Covid-19 experience. There were statistically significant differences in general work stress, hopelessness, and job satisfaction for single health-care staff compared with those who were married (35.41 vs. 40.66).

**Table 1:** Demographic characteristics of the sample and descriptive characteristics of scales (*N* = 416).

		General work stress		Suicide cognitions		Hopelessness		Job satisfaction	
Variable	Level	*M*	SD	*M*	SD	*M*	SD	*M*	SD
Gender	Female	23.28	9.38	38,54	17,47	7,05	3,88	13,19	3,87
	Male	23.31	9.28	37,88	17,51	6,72	3,74	13,42	4,34
	Test (*t*-test)	*t* (1, 414) = −0.029		*t* (1, 414) = 0.350		*t* (1, 414) = 0.779		*t* (1, 414) = −0.529	
Marital status	Married	22,97	9,32	35,41	16,78	7,03	3,60	13,33	4,03
	Single	23,69	9,37	40,66	17,68	6,85	4,13	13,16	3,99
	*t*-test	*t* (1, 414) = 0.433		*t* (1, 414) = 0.002**		*t* (1, 414) = 0.625		*t* (1, 414) = 0.685	
Socioeconomic status	Low	26,90	10,11	47,46	19,36	7,67	3,98	12,45	4,45
	Average	22,2,296	8,57	35,74	15,01	6,74	3,67	13,54	3,82
	High	17,6,923	7,94	23,42	12,21	5,80	4,47	14,07	3,28
	Test (ANOVA)	*F* (2, 413) = 16.503**		*F* (2, 413) = 33.346**		*F* (2, 413) = 3.700*		*F* (2, 413) = 3.688**	
Health-care workers	Doctor	23,51	8,15	34,82	15,04	6,71	3,81	13,30	4,25
	Nurse	23,24	9,65	39,41	18,54	7,07	3,77	13,41	4,06
	Others	23,18	9,87	39,59	16,78	6,94	4,10	12,81	3,6
	Test (ANOVA)	*F* (2, 413) = 0.035		*F* (2, 413) = 2.686		*F* (2, 413) = 0.307		*F* (2, 413) = 0.737	
Working hours	<6 h	18,42	7,99	21,3	8,70	6,19	4,41	14,25	3,81
	6–9 h	22,75	8,66	35,97	11,35	6,65	3,42	13,40	3,42
	10–12 h	26,73	9,64	49,81	15,86	7,59	3,49	13,45	4,69
	>12 h	30,38	8,71	67,59	14,11	9,26	4,73	9,47	4,25
	Test (ANOVA)	*F* (2, 412) = 20.737**		*F* (2, 412) = 147.636**		F (2, 412) = 6.677**		*F* (2, 412) = 12.983**	
COVID-19 experience	Infected	22,76	9,25	38,61	17,3	6,84	3,92	13,44	3,93
	Non-infected	24,58	9,48	37,75	17,93	7,24	3,66	12,82	4,19
	Test (*t*-test)	*t* (1, 414) = 0.070		*t* (1, 414) = 0.648		*t* (1, 414) = 0.338		*t* (1, 414) = 0.150	

*p < 0.05; **p < 0.01; ***p < 0.001.

One-way ANOVA was used to examine general work stress, suicidal cognitions, hopelessness, and job satisfaction by socioeconomic status, occupation, and working hours (see Table 1). There were statistically significant differences between group means concerning socioeconomic status and working hours, while there were no differences in occupation. In order to reveal the source of the difference, a Tukey post-hoc test was conducted. The results indicated that low (26.90 ± 10.11), and average socioeconomic status (22.23 ± 8.57) had more general work stresses than high socioeconomic status (17.69 ± 7.95). Low (47.46 ± 19.36), and average socioeconomic status (35.74 ± 15.01) had more suicide cognitions than high socioeconomic status (23.42 ± 12.22). Moreover, low socioeconomic status (7.67 ± 3.99) had more hopelessness than high socioeconomic status (5.80 ± 4.47). To detect the difference of source for working hours, a Tukey *post hoc* test was conducted. According to the results, the general work stress of healthcare workers who work over 12 h (30.38 ± 8.70), between 10 and 12 h (26.73 ± 9.64), and between 6 and 9 h (22.74 ± 8.66) was significantly higher than that of those who work less than 6 h (18.42 ± 7.99). The level of suicide cognitions of health care workers working more than 12 h (67.59 ± 14.10), between 9 and 12 h (49.80 ± 15.85), between 6 and 9 h (35.97 ± 11.34), was significantly higher than those working less than 6 h (21.29 ± 8.69). The hopelessness of health-care workers working more than 12 h (9.26 ± 4.25), was significantly higher than those working less than 6 h (6.16 ± 4.40). Besides, the level of job satisfaction of health-care workers working less than 6 h (14.25 ± 3.80), was significantly higher than those working more than 12 h (9.47 ± 4.25).

Table 2 presents descriptive statistics and correlation coefficients among the variables included in the study. These variables’ skewness and kurtosis values fall within the acceptable normal distribution range of the proposed threshold value of ±2; therefore, we did not find evidence that the normality assumption had been violated (73). The correlation analysis revealed that general work stress positively and significantly negatively correlated with suicide cognitions and hopelessness, indicating that one variable changes in the same direction as the other. All variables were found to be either low or moderately correlated, according to the study results.

**Table 2:** The descriptive statistics and correlations between the variables (*N* = 416).

Variable	1.	2.	3.	4.
1. General work stress	—			
2. Suicide cognitions	0.50^**^	—		
3. Hopelessness	0.20^**^	0.33^**^	—	
4. Job satisfaction	−0.35^**^	−0.31^**^	−0.24^**^	—
Mean	23.29	38.35	6.96	13.26
Std. Deviation	9.34	17.47	3.84	4.01
Skewness	0.39	0.61	0.70	0.07
Kurtosis	−0.33	−0.36	0.87	0.30

**p < 0.05.

### Mediation analysis

**Figure 2:**
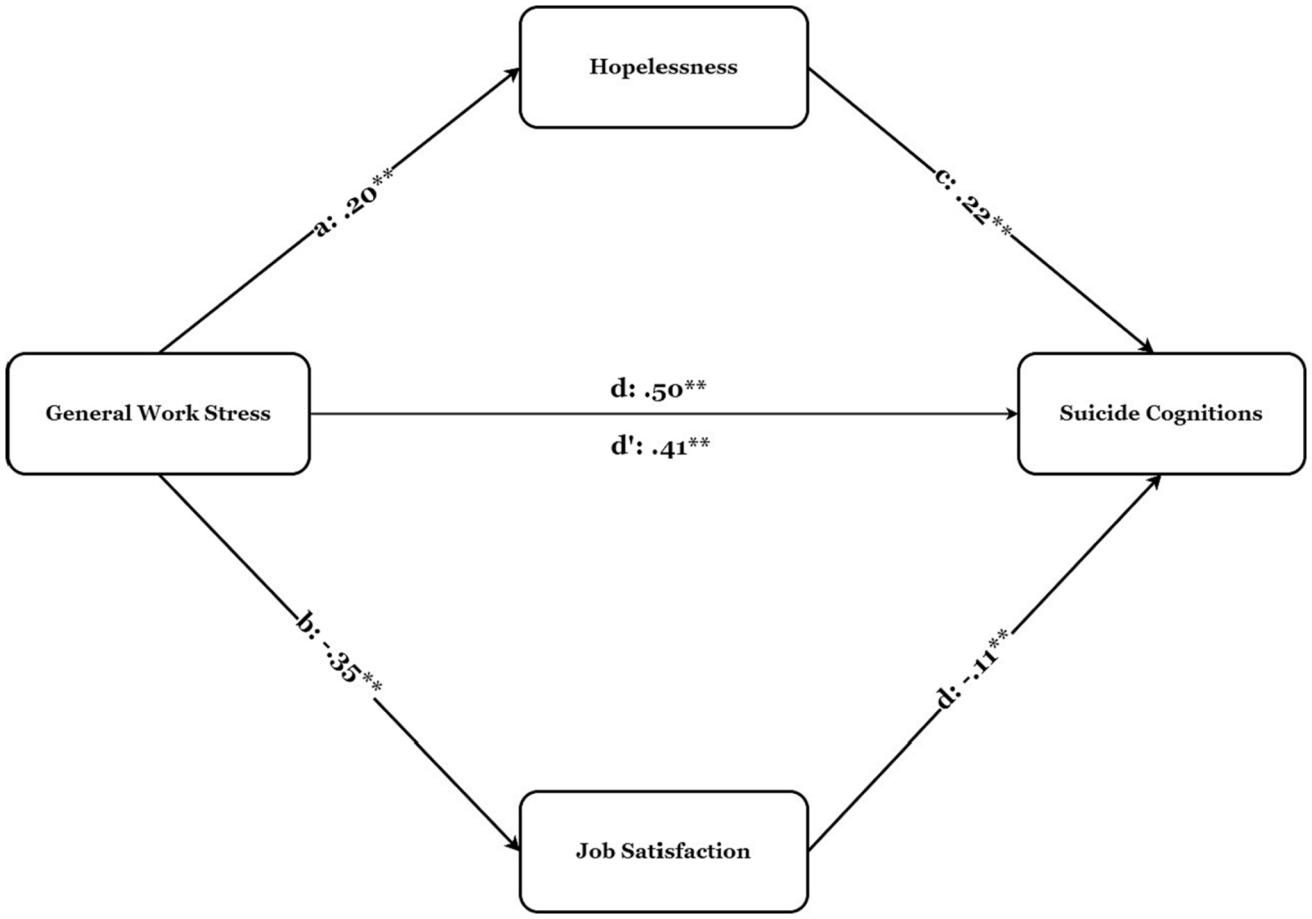
Parallel mediation model showing path coefficients of the proposed model.

The mediation analysis results are presented in Table 2 and Figure 2. A direct relationship of general work stress on suicide cognitions (total, *β* = 0.50, *p* < 0.001) was found. General work stress also had a positive relationship with hopelessness (direct effect, *β* = 0.20, *p* < 0.001). It was also a negative relationship with job satisfaction (direct, *β* = −0.35, *p* < 0.001). Hypothesis 1 was confirmed based on the results obtained. Path coefficients were examined to examine the relationship between hopelessness and suicide cognitions, and the results indicated that hopelessness had a relationship with suicide cognitions (direct, *β* = 0.22, *p* < 0.001). Moreover, job satisfaction also had a relationship with suicide cognitions (direct, *β* = −0.11, *p* < 0.001). Hypothesis 2 was confirmed based on the results obtained. The results showed that this coefficient remained significant when mediators were included in the analysis (i.e., hopelessness and job satisfaction) (direct, *β* = 0.41, *p* < 0.001). General work stress had a significant indirect relationship with suicide cognitions through hopelessness [indirect = 0.08, SE = 0.03, 95% CI = (0.03, 0.13)]. Furthermore, General work stress had a significant indirect relationship with suicide cognitions through job satisfaction [indirect = 0.07, SE = 0.03, 95% CI = (0.01, 0.13)]. The results indicated that the relationship between general work stress and suicide cognitions was parallelly mediated by hopelessness and job satisfaction. Hypothesis 3 was confirmed based on the results obtained (Table 3).

**Table 3:** Statistical significance of the variables and their path coefficients.

				95% C.I.				
Path	Effect	Coefficient	SE	Lower	Upper	*β*	*z*	*p*
Indirect	GWSS ⇒ BHS ⇒ SCS	0.08	0.03	0.03	0.13	0.04	3.30	<0.001^**^
	GWSS ⇒ JSS ⇒ SCS	0.07	0.03	0.01	0.13	0.04	2.38	<0.05^*^
Components	GWSS ⇒ JSS	−0.15	0.02	−0.19	−0.11	−0.35	−7.63	<0.05^*^
	JSS ⇒ SCS	−0.47	0.19	−0.84	−0.10	−0.11	−2.50	<0.001^**^
	GWSS ⇒ BHS	0.08	0.02	0.04	0.12	0.20	4.20	<0.001^**^
	BHS ⇒ SCS	1.01	0.19	0.64	1.38	0.22	5.34	<0.001^**^
Direct	GWSS ⇒ SCS	0.77	0.09	0.61	0.93	0.41	9.33	<0.001^**^
Total	GWSS ⇒ SCS	0.92	0.08	0.77	1.08	0.50	11.63	<0.001^**^

**p < 0.001; *p < 0.05; SE, standard error; GWSS, general work stress; SCS, suicide cognitions; BHS; hopelessness; JSS, job satisfaction.

## Discussion

This study aimed to explore the influence of general work stress on suicide cognitions and its potential mediating mechanisms of job satisfaction and hopelessness. As hypothesized, the results of this study demonstrated that general work stress significantly and negatively predicts job satisfaction, while it significantly and positively predicts hopelessness and suicide cognitions. This confirms the first research hypothesis. These results are consistent with the results of previous studies, showing the positive associations between general work stress and suicide cognition (74–76). Considerable job-related stress and are more prone to exhibit a variety of psychological disorders, such as PTSD and suicidal thoughts (77). The greatest rates of psychological distress were recorded among nurses, women workers, frontline health-care workers, younger medical personnel, and employees in locations with higher infection rates, according to a systematic analysis analyzing the mental health concerns among health-care workers after the pandemic (78). Related research has proposed a 7-factor model linking PTSD to elevated suicide risk (79). Another research found that health-care workers were more likely to have mental health problems after exposure to long and irregular work hours (80). In the study of Rahman and Plummer (81), factors associated with nurses’ mental stress and the consequences of suicide were identified. These studies’ findings indicate a strong association between general work stress and suicide cognition.

Researchers examined the path coefficients between hopelessness and suicidal cognitions and found that the former was significantly significant. Accordingly, hopelessness had a mediating effect on the relationship between general work stress and suicide cognitions. The findings support the second hypothesis. When a person engages in ruminative, negative thought patterns, they are more likely to develop hopelessness or suicide cognitions (82, 83), and suicide attempts among those suffering from depression are frequently triggered by hopelessness (84). Therefore, suicide-specific (e.g., poor life-affirming) cognitions may be important in figuring out the associations between depression and despair and suicidal thoughts and actions (85). Although hopelessness and suicide cognitions are positively correlated, some studies have shown that certain practices can boost job satisfaction and reduce negative thoughts, including suicide cognitions (86, 87).

The coefficient remained statistically significant even after adding hopelessness and job satisfaction as mediators. In terms of suicidal cognitions, hopelessness was a major factor associated with general work stress. Moreover, job satisfaction was a strong mediator between general work stress and suicidal ideation. Both feelings of hopelessness and job satisfaction were found to mediate the link between general work stress and suicidal thoughts. As a result of the analysis, the third hypothesis is supported. Consequently, health-care workers with higher levels of work stress and hopelessness but lower levels of job satisfaction tend to have more suicide cognitions. This can lead to a decrease in focus and concentration, as well as productivity and efficiency. It can also lead to increased negative emotions such as anxiety and depression. Ultimately, this can affect health-care workers’ quality of care. Much recent research revealed the relationship between stress and suicide cognitions (88–90), and there are studies giving the association between suicide cognitions and negative thoughts like hopelessness (91–93).

## Implications

The present study significantly advances our understanding of the relationship between general work stress and suicide cognitions by showing the mediating roles played by hopelessness and job satisfaction in this relationship. The findings of this study demonstrated the pivotal significance of hopelessness and job satisfaction in dealing with the mental well-being of health-care workers within the workplace context. General work stress increases hopelessness and reduces job satisfaction, which in turn increases suicide cognitions. As higher hopelessness and lower job satisfaction were associated with higher general work stress and suicide cognitions, it is important that hospitals tailor training programs to improve the capacity of health-care workers to effectively cope with stressors and provide better care for patients. The results highlight the need for hospitals and health-care institutions for tailored training programs. These programs should aim to contribute to the coping mechanisms of health-care workers, enabling them to deal with stressors effectively and deliver better care for patients. Such training interventions can be executed through diverse ways, including both conventional face-to-face sessions and contemporary virtual platforms, including social media channels, webinars, and video technologies.

## Limitations

While this study enhances our understanding of the associations between general work stress, suicide cognitions, hopelessness, and job satisfaction, it is not exempt from limitations. The cross-sectional design restricts our ability to establish causality among the variables. To address this, future studies could benefit from incorporating longitudinal designs by collecting data at multiple time points to account for dynamic processes influencing the results. Furthermore, the sample demographic characteristics, including age, gender, and socioeconomic status, may have introduced confounding variables. It is important to consider these variables when interpreting the findings. Further research is warranted to validate the results. Additionally, the study relied on online survey data collection, which is susceptible to selection bias and exclusion of participants without internet access due to factors like affordability and accessibility. Therefore, generalizing these findings to the entire population may be challenging. Future studies should aim for a more representative sample, ensuring equal gender representation among health-care workers.

### Conclusion

In conclusion, this study contributes to the growing body of literature indicating that various psychological factors, both positive and negative, including hopelessness and job satisfaction, play important roles in influencing suicide cognitions among health-care workers. These findings hold implications for the development and implementation of targeted interventions aimed at addressing factors associated with suicide cognitions. Therefore, these results underscore the significance of hospital-based prevention and intervention services designed to mitigate hopelessness, enhance job satisfaction, and consequently, alleviate the impact of general work stress and suicide cognitions.

## Data availability statement

The raw data supporting the conclusions of this article will be made available by the authors, without undue reservation.

## Ethics statement

The studies involving humans were approved by the Ağrı İbrahim Çeçen University Ethics Committee. The studies were conducted in accordance with the local legislation and institutional requirements. The participants provided their written informed consent to participate in this study.

## Author contributions

AS: Writing – original draft, Writing – review & editing, Project administration. AK: Project administration, Writing – original draft, Writing – review & editing, Conceptualization, Data curation, Formal analysis, Investigation, Methodology, Resources, Software. IA: Writing – review & editing. MY: Conceptualization, Writing – original draft, Writing – review & editing, Supervision, Validation. HÖ: Conceptualization, Data curation, Software, Writing – review & editing. FC: Writing – review & editing. SZ: Funding acquisition, Writing – review & editing.

## Funding

This work was supported by the Italian Ministry of Health with Current Research Funds.

## Footnotes

^1^Rudd MD, Schmitz B, McClenen R, Joiner T, Elkins G, Claassen C. The suicide cognitions scale: a suicide-specific measure of hopelessness. J Abnorm Psychol. (unpublished).
